# Powerful anti-tumor and anti-angiogenic activity of a new anti-vascular endothelial growth factor receptor 1 peptide in colorectal cancer models

**DOI:** 10.18632/oncotarget.3384

**Published:** 2015-03-25

**Authors:** Valeria Cicatiello, Ivana Apicella, Laura Tudisco, Valeria Tarallo, Luigi Formisano, Annamaria Sandomenico, Younghee Kim, Ana Bastos-Carvalho, Augusto Orlandi, Jayakrishna Ambati, Menotti Ruvo, Roberto Bianco, Sandro De Falco

**Affiliations:** ^1^ Angiogenesis Lab, Institute of Genetics and Biophysics “Adriano Buzzati-Traverso” – CNR, Naples, Italy; ^2^ Bio-Ker, MultiMedica Group, Napoli, Italy; ^3^ Medical Oncology, Department of Clinic Medicine and Surgery, University of Naples “Federico II”, Italy; ^4^ Institute of Biostructures and Bioimaging – CNR and CIRPeB, University of Naples “Federico II”, Italy; ^5^ Department of Ophthalmology & Visual Sciences, University of Kentucky, Lexington, KY, USA; ^6^ Anatomic Pathology Institute, Department of Biomedicine and Prevention, Tor Vergata University of Rome, Italy; ^7^ IRCCS MultiMedica, Milan, Italy

**Keywords:** colorectal cancer, VEGFR1, angiogenesis, metastasis, choroid neovascularization

## Abstract

To assess the therapeutic outcome of selective block of VEGFR1, we have evaluated the activity of a new specific antagonist of VEGFR1, named iVR1 (inhibitor of VEGFR1), in syngenic and xenograft colorectal cancer models, in an artificial model of metastatization, and in laser-induced choroid neovascularization. iVR1 inhibited tumor growth and neoangiogenesis in both models of colorectal cancer, with an extent similar to that of bevacizumab, a monoclonal antibody anti-VEGF-A. It potently inhibited VEGFR1 phosphorylation *in vivo*, determining a strong inhibition of the recruitment of monocyte-macrophages and of mural cells as confirmed, *in vitro*, by the ability to inhibit macrophages migration. iVR1 was able to synergize with irinotecan determining a shrinkage of tumors that became undetectable after three weeks of combined treatment. Such treatment induced a significant prolongation of survival similar to that observed with bevacizumab and irinotecan combination. iVR1 also fully prevented lung invasion by HCT-116 cells injected in mouse tail vein. Also, iVR1 impressively inhibited choroid neovascularization after a single intravitreal injection. Collectively, data showed the strong potential of iVR1 peptide as a new anti-tumor and anti-metastatic agent and demonstrate the high flexibility of VEGFR1 antagonists as therapeutic anti-angiogenic agents in different pathological contexts.

## INTRODUCTION

The development and maintenance of functional vessels through angiogenesis and arteriogenesis processes in pathological conditions require the cooperation of several growth factor families, of multiple cell types and the presence of certain conditions such as hypoxia and inflammation [1, 2]. The family of vascular endothelial growth factors (VEGF) and related receptors play a central role in promoting vessel formation and maturation. Indeed, since a decade they are the only validated targets for the anti-angiogenesis therapy in cancer and ocular neovascular diseases [3, 4]. After the approval by FDA of bevacizumab (Avastin), a humanized monoclonal antibody (mAb) against VEGF-A for the treatment of metastatic colorectal carcinoma (mCRC) in combination with chemotherapy [5], other anti-angiogenic drugs targeting members of VEGF family have been developed such as aflibercept, a recombinant fusion protein consisting of VEGF-binding extracellular domains of VEGFR1 and VEGFR2 fused to the Fc portion of human IgG1, and several multitargets tyrosine kinase inhibitors which inhibit also VEGF receptors [4].

Anti-angiogenic drugs have been approved to treat different types of cancer, such as mCRC, non-small cell lung carcinoma, metastatic renal cell carcinoma, gastrointestinal stromal tumor, hepatocellular carcinoma, and others. However, despite the significant clinical success of anti-angiogenic therapies, alternative strategies are desirable to improve their therapeutic efficacy and to overcome inherent/acquired resistance and the relevant side effects [6, 7].

VEGF receptor 1 (VEGFR1, also known as Flt-1) is the common receptor for the three pro-angiogenic family members VEGF-A, VEGF-B, and placental growth factor (PlGF). It is the unique receptor for VEGF-B and PlGF, and shows higher affinity for VEGF-A compared to VEGFR-2 (also known as KDR or Flk-1), which is exclusively recognized by VEGF-A. VEGFR1 expression is upregulated by hypoxia and is also expressed as soluble form generated by an alternative splicing (termed sVEGFR1), which is one of the most potent physiological inhibitor of angiogenesis [8].

Differently from VEGFR2 found almost exclusively in endothelial cells, VEGFR1 is expressed in several types of cells, many of which play a fundamental role during the angiogenesis and arteriogenesis process. Endothelial cells, bone marrow stem/precursor cells, circulating endothelial cells, stromal cells, perycites and smooth muscle cells, monocyte-macrophages, dendritic cells, all express functional VEGFR1 that once activated induces mainly survival, proliferation and migration pathways [9]. In addition, several human cancer cells express VEGFR1 [10].

VEGFR1 was initially classified as decoy receptor for VEGF-A because of its low tyrosine kinase activity, compared to VEGFR2 [11], and the viability of mice in which VEGFR1 tyrosine kinase domain was ablated (*Flt1^tk−/−^*) [12]. However several other reports re-evaluated it as a functional receptor, thereby as possible therapeutic target. *Flt1^tk−/−^* mice showed impaired angiogenesis and reduced inflammation under several disease conditions, such as arthritis, atherosclerosis, choroidal neovascularization and cancer growth [13–15]. The activation of VEGFR1 promotes tumor angiogenesis and metastasis through diverse mechanisms [16]. It stimulates angiogenesis by recruiting endothelial and monocyte progenitor cells from bone marrow into tumor vasculature [17, 18] as well as smooth muscle cells to cover and stabilize neovessels [19]. VEGFR1 also plays a central role in the modulation of inflammatory component of tumors, driving the recruitment and activity of macrophages and dendritic cells and contributing to tumor-cell survival during the epithelial–mesenchymal transition [20]. Furthermore, VEGFR1 activation markedly promotes pulmonary metastases through induction of matrix metalloproteinase-9 secretion [21] and plays a crucial role in the establishment of pre-metastatic niches [22].

The functional role of VEGFR1 in tumor and metastasis contexts was confirmed using inhibitors from different sources. Ribozyme [23], mAb [24], peptides [25, 26], or DNAzyme [27] specifically targeting VEGFR1, all inhibit tumor growth and metastasis formation.

Here, we describe the potent anti-angiogenic, anti-tumor, and anti-metastatic activity of a tetrameric tripeptide named iVR1 (inhibitor of VEGFR1), which is able to specifically bind mouse and human VEGFR1 blocking receptor activation by preventing the interaction of the natural ligands VEGF-A, VEGF-B, PlGF and VEGF-A/PlGF heterodimer (IC_50_ 6–10 μM) [28].

The anti-angiogenic activity of iVR1 has been first assessed in the choroid neovascularization (CNV) model. Then, iVR1 activity has been assayed in syngenic and xenograft models of colorectal cancer and compared to that of mAbs inhibiting the two main ligands of VEGFR1, VEGF-A and PlGF. The ability of iVR1 to synergize with chemotherapy, as well as the anti-metastatic properties, evaluating lung invasion by colorectal cancer cells injected in the blood circulation, have been also investigated.

## RESULTS

### Anti-angiogenic *in vivo* activity of iVR1

**Figure 1:**
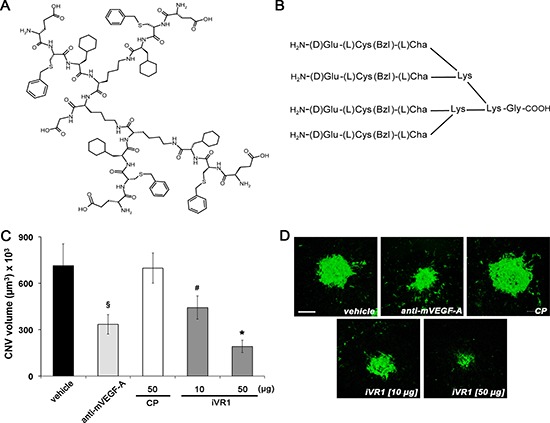
Anti-angiogenic activity of iVR1 *in vivo* **(A)** Chemical structure of iVR1 tetrameric tripeptide that has a calculated molecular mass of 2362.02 g/mol. **(B)** Schematic representation of the iVR1. L-Cys(Bzl): L-cysteine(S-benzyl); L-Cha, L-cyclohexylalanine. **(C)** iVR1 inhibited laser-induced CNV in a dose-dependent manner, whereas CP was ineffective. CNV volumes were measured by confocal evaluation of Isolectin B4 staining of RPE-choroid flat mounts. Data are represented as the mean ± SEM (*N* = 8). *p < 0.0005 and ^§^*p* < 0.01 compared to vehicle and CP; ^#^*p* < 0.05 versus CP. **(D)** Representative pictures of CNV flat mounts. Scale bar: 100 μm.

iVR1, previously referred as 4.23.5, has a molecular mass of 2362.02 g/mol and is composed by the tripeptide H2N-D-Glu–L-Cys(Bzl)–L-Cha, where D-Glu is D-glutammic acid, L-Cys(Bzl) is L-cysteine-S-benzyl and L-Cha is L-cyclohexylalanine, engrafted on a tri-lysine core (Figure 1A, 1B). The *in vitro* activity of iVR1 has been yet fully characterized. The presence of unnatural amino acids and the multimeric structure confer high resistance to degradation in biological fluids. It specifically binds VEGFR1 and does not interfere with VEGFR2 activity. It prevents both the VEGFR1 phosphorylation and the capillary-like tube formation of human primary endothelial cells, as well as neovascularization of chicken embryo chorioallantoic membrane induced by PlGF or VEGF-A [28].

In order to assess the iVR1 anti-angiogenic activity *in vivo*, we used the laser-induced CNV model. Immediately after the induction of laser damage, single intravitreal injections of iVR1, of the control peptide (CP) and of anti-mouse VEGF-A polyclonal antibody, were performed. After seven days, CNV volume was evaluated by immunofluorescence analysis of retinal pigment epithelium (RPE) choroid flat mounts. CP (50 μg, 21 nmol) was unable to inhibit CNV whereas, as expected, anti-mouse VEGF-A induced a strong and significant inhibition compared to vehicle and CP (−52.5% in average). iVR1 was able to induce a dose-dependent inhibition with an impressive reduction of CNV (−73.0% in average versus vehicle and CP) at the highest concentration delivered (50 μg, 21 nmol) (Figure 1C, 1D).

### iVR1 inhibited syngeneic and xenograft colorectal cancer growth and neovascularization

**Figure 2:**
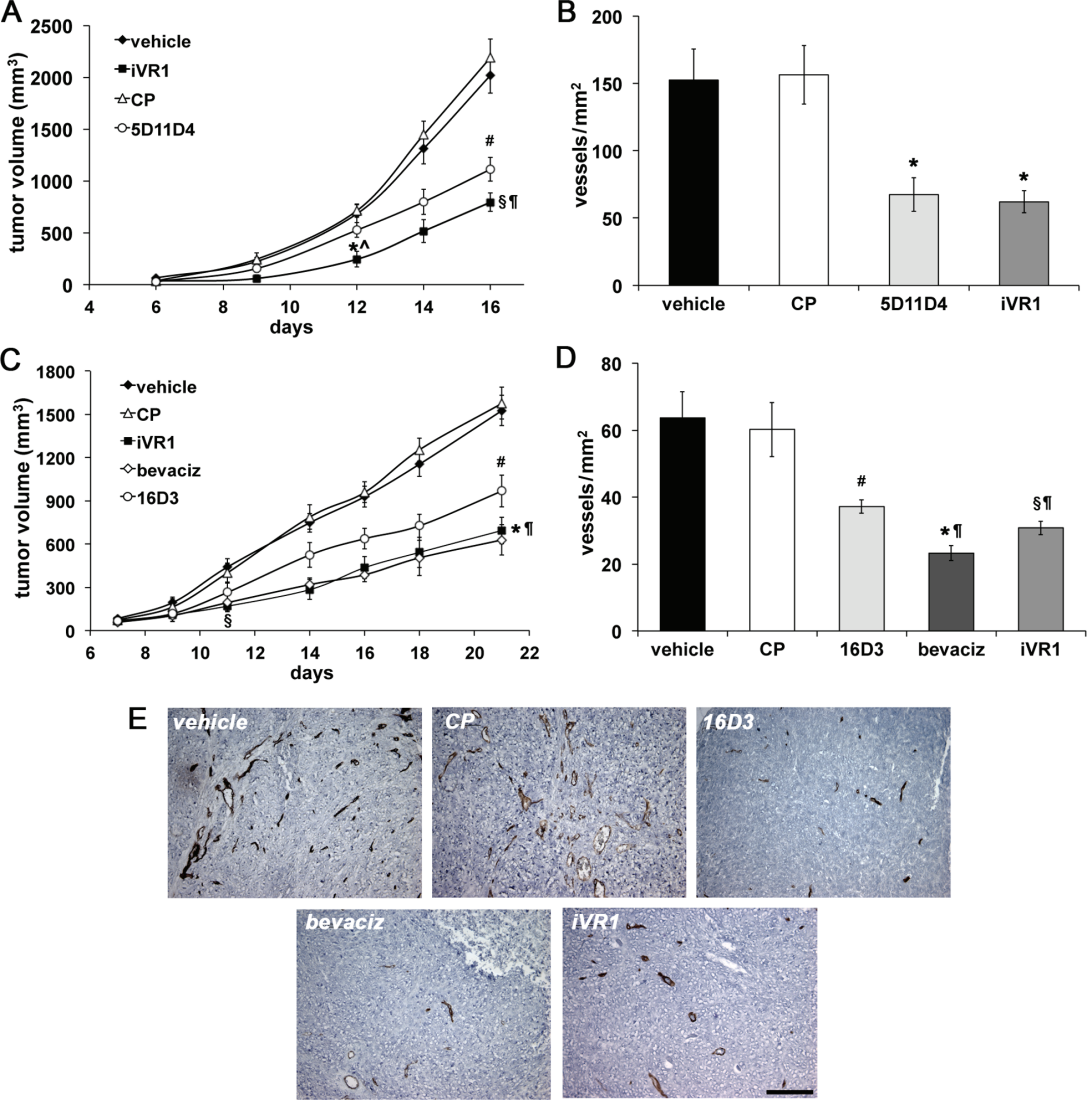
iVR1 inhibited growth and neo-angiogenesis of syngenic and xenograft colorectal tumors CT26 mouse colon carcinoma cells **(A)** or HCT-116 human colorectal cancer cells **(C)** were injected subcutaneously in Balb/c mice or CD1 nude athymic mice, respectively. iVR1 and control peptide (CP) were delivered at 50 mg/kg each other day. Anti-PlGF mAbs 5D11D4 (mouse) and 16D3 (human) were delivered at 25 mg/kg twice a week. Bevacizumab (bevaciz) was delivered at 5 mg/kg, twice a week. Vehicle was PEG400/water 1:1. TV was measured three times a week and data are represented as the mean ± SEM (*N* = 7). A, *p < 0.001 and ^§^*p* < 0.0001 versus vehicle and CP; ^*p* < 0.02 and ^¶^*p* < 0.05 vs 5D11D4; #*p* < 0.002 versus vehicle and CP. C, ^§^*p* < 0.01 and *p = 0.0001 versus vehicle and CP, ^¶^*p* < 0.05 versus 16D3; ^#^*p* = 0.0027 versus vehicle and CP. Vessel density of syngenic **(B)** and xenograft **(D)** tumors, were calculated analyzing five optical fields for each tumor, counting CD31-positive vessels (brown). Data are represented as the mean ± SEM. B, *p < 0.007 versus vehicle and CP. D, ^§^*p* < 0.005, ^#^*p* < 0.02, and *p < 0.0005, versus vehicle and CP. ^¶^*p* < 0.05 bevaciz versus iVR1 and CP and iVR1 versus 16D3. **(E)** Representative pictures of CD31 staining (brown) of HCT-116 tumors. Scale bar, 100 μm.

Syngenic colon carcinomas were generated in Balb/c mice by subcutaneous injection of CT26 cells. After six days the treatments with vehicle, anti-mouse PlGF monoclonal antibody (mAb) 5D11D4 [29], iVR1 and the control peptide (CP) started [28]. iVR1-treated mice showed a strong and significant reduction of tumor growth starting from six days of treatment, compared to vehicle and CP, and also compared to mAb 5D11D4, which was also able to inhibit tumor growth but with less efficacy (Figure 2A). Tumor growth reduction paired with inhibition of tumor neoangiogenesis, as assessed by vessel density measurement of tumors explanted 16 days after cell inoculation. iVR1 and 5D11D4 were able to induce a significant and similar inhibition of blood vessel density compared to vehicle and CP (−59.8% and −55.3% in average, respectively) (Figure 2B).

To generate tumor xenografts, we injected the HCT-116 colorectal cancer cells in athymic nude mice and, after seven days, treatments with bevacizumab, anti-human PlGF mAb 16D3, iVR1 and CP peptides started (Figure 2C). Surprisingly, tumor growth curves in iVR1 and bevacizumab treated mice were fully superimposable, resulting in a significant tumor growth delay starting from four days of treatment, compared to vehicle and CP. The mAb 16D3, able to block only PlGF produced by human cells, also determined a significant inhibition compared to vehicle and CP. Bevacizumab and iVR1 tumor growth inhibitions were also significantly higher compared to 16D3 at the end of treatments (Figure 2C). The analysis of vessel density performed on tumors explanted 21 days after cell inoculation (Figure 2D, 2E) showed that iVR1 determined a strong inhibition of neovessel formation (−50.7% on average), greater than that afforded with 16D3 (−39.8% on average), and slightly lower of that induced by bevacizumab (−62.4% on average), when compared to vehicle and CP.

Collectively, these data demonstrated that iVR1 is a potent anti-tumor and anti-angiogenic molecule, with an efficacy similar to that displayed by bevacizumab and greater than that of mAbs anti-PlGF assayed.

### iVR1 is a potent inhibitor of monocyte-macrophages and mural cells recruitment

**Figure 3:**
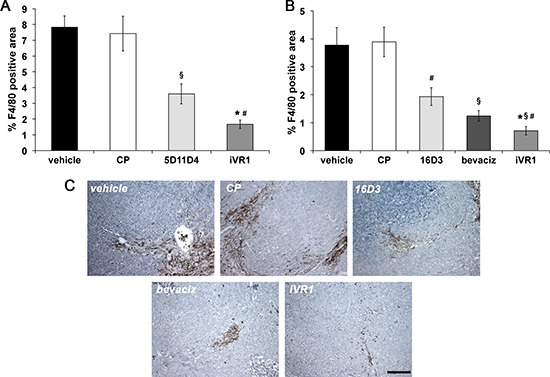
iVR1 inhibited the recruitment of monocyte-macrophages in syngenic and xenograft colorectal tumors The area of monocyte-macrophage infiltrate in syngenic **(A)** and xenograft **(B)** tumors was determined by immunostaining with anti-F4/80 antibody and calculated on five optical fields for each tumor. Data are represented as the mean ± SEM (*N* = 7). A, *p < 0.0005 and ^§^*p* < 0.01 versus vehicle and CP; ^#^*p* < 0.002 versus 5D11D4. B, *p < 0.0005, ^§^*p* < 0.005 and ^#^*p* < 0.05, versus vehicle and CP; ^#^*p* < 0.05 versus bevacizumab (bevaciz); ^§^*p* < 0.005 versus 16D3 (anti human PlGF mAb). **(C)** Representative pictures of F4/80 staining (brown) of HCT-116 tumors. Scale bar, 100 μm.

Monocytes-macrophages play a well-established role in tumor angiogenesis [30, 31], and VEGFR1 has an active role for their recruitment at neo-angiogenic sites. We thereby determined the extent of monocytes-macrophages infiltration in tumors by F4/80 immunohistochemical analysis. In syngenic tumors, mAb 5D11D4 determined a significant reduction of F4/80 positive area compared to vehicle and CP (−52.7% on average), as expected by blocking one of theVEGFR1 specific ligands. The block of all the VEGFR1 ligands by iVR1 determined a greater reduction of F4/80 positive area compared to vehicle and CP (−78.0% on average). Such reduction was also significantly higher compared to 5D11D4 (−53.6%) (Figure 3A). In tumor xenografts, iVR1 treatment induced an impressive suppression of monocyte-macrophages recruitment compared to vehicle and CP (−81.3% on average). The inhibition resulted considerably higher compared to that produced by bevacizumab or 16D3 treatments, which, however, also induced a substantial inhibition with respect to vehicle and CP (−67.6% and −49.4%, on average) (Figure 3B, 3C).

**Figure 4:**
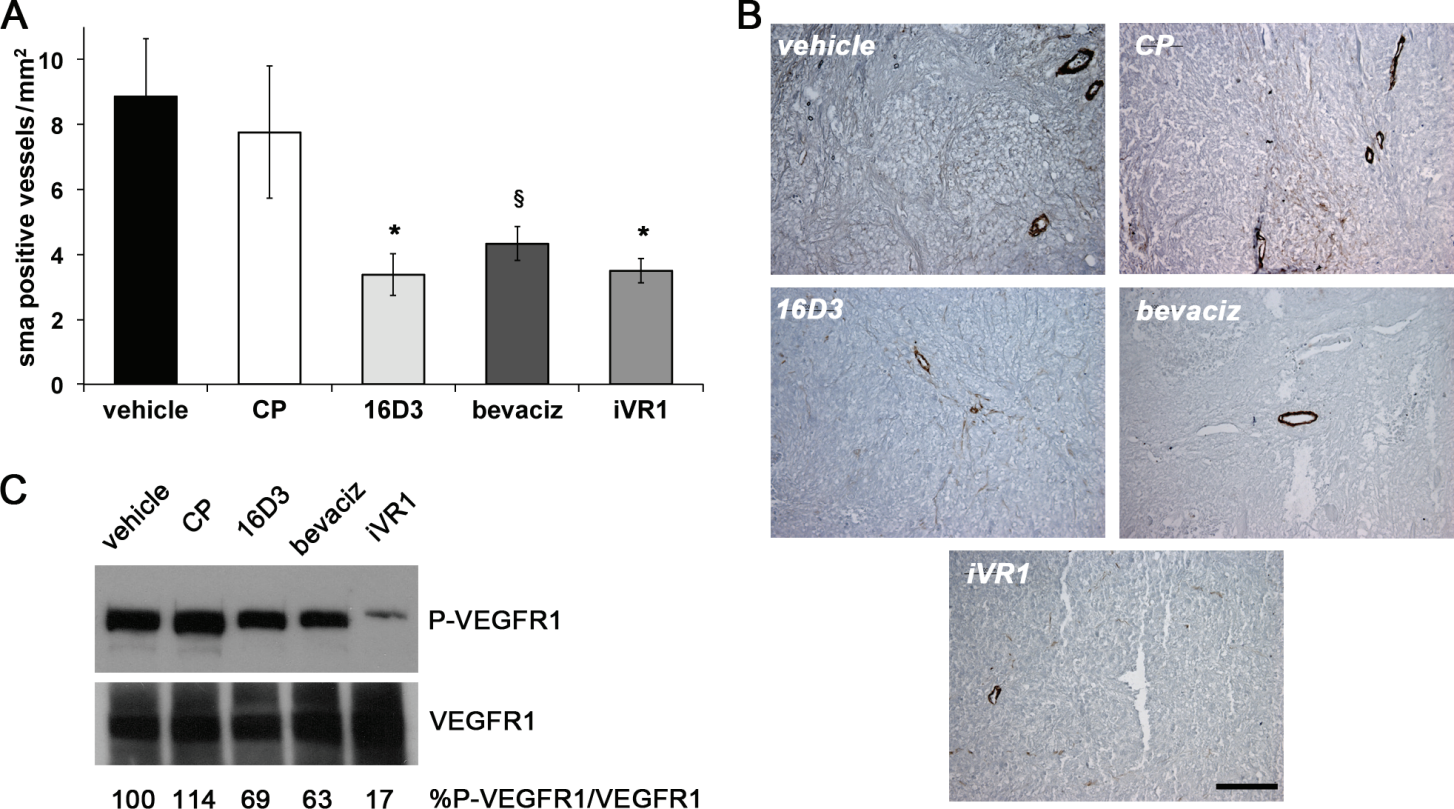
iVR1 inhibited the recruitment of smooth muscle cells in xenograft colorectal tumors blocking VEGFR1 activation **(A)** Smooth muscle α-actin (SMA)-positive vessels were counted on five optical fields for each tumor to quantify density of vessels covered by SMCs. *p < 0.020 and ^§^*p* < 0.05 versus vehicle and CP. **(B)** Representative pictures of SMA staining (brown) of HCT-116 tumors. Scale bar, 100 μm. **(C)** Top, western blot analysis of VEGFR1 phosphorylation performed on mixed matching amounts of protein extracts belonging to the same experimental tumor group. Low, normalization with anti-VEGFR1 antibody performed on the same filter. The values of densitometry analyses are shown. Values (in percentages) were calculated as the ratio of degree of receptor phosphorylation with respect to the total receptor amounts. The value of 100 has been arbitrarily assigned to vehicle.

VEGFR1 has also a functional role in the recruitment of mural cells, a step required for maturation and stabilization of neovessels [19]. The extent of vessel coverage by smooth muscle cells in tumor xenografts was estimated by immunostaining with anti-smooth muscle α-actin (SMA). All the treatments determined significant and extensive reduction of vessel coverage, similar for 16D3 and iVR1 (−59.3% and −58.0% on average, respectively) and slightly lower for bevacizumab (−51% on average) compared to vehicle and CP (Figure 4A, 4B).

VEGFR1 phosphorylation status was evaluated in tumor xenograft protein extracts. Matching amounts of protein extracts from tumors belonging to the same group were mixed and analyzed by western blot. The block of single ligands, achieved with bevacizumab or 16D3, determined a partial decrease of VEGFR1 phosphorylation (−35.2% and −22.6% on average, respectively) compared to vehicle and CP, which were both ineffective. iVR1 induced a powerful lessening of VEGFR1 phosphorylation (−78.2% on average, versus vehicle and CP)(Figure 4C).

Collectively, these data demonstrated that iVR1 is a potent inhibitor of VEGFR1 activation *in vivo* and of VEGFR1-mediated recruitment of non-endothelial cells involved in tumor angiogenesis.

### iVR1 inhibited cell migration and tumor xenografts growth in a dose-dependent manner

**Figure 5:**
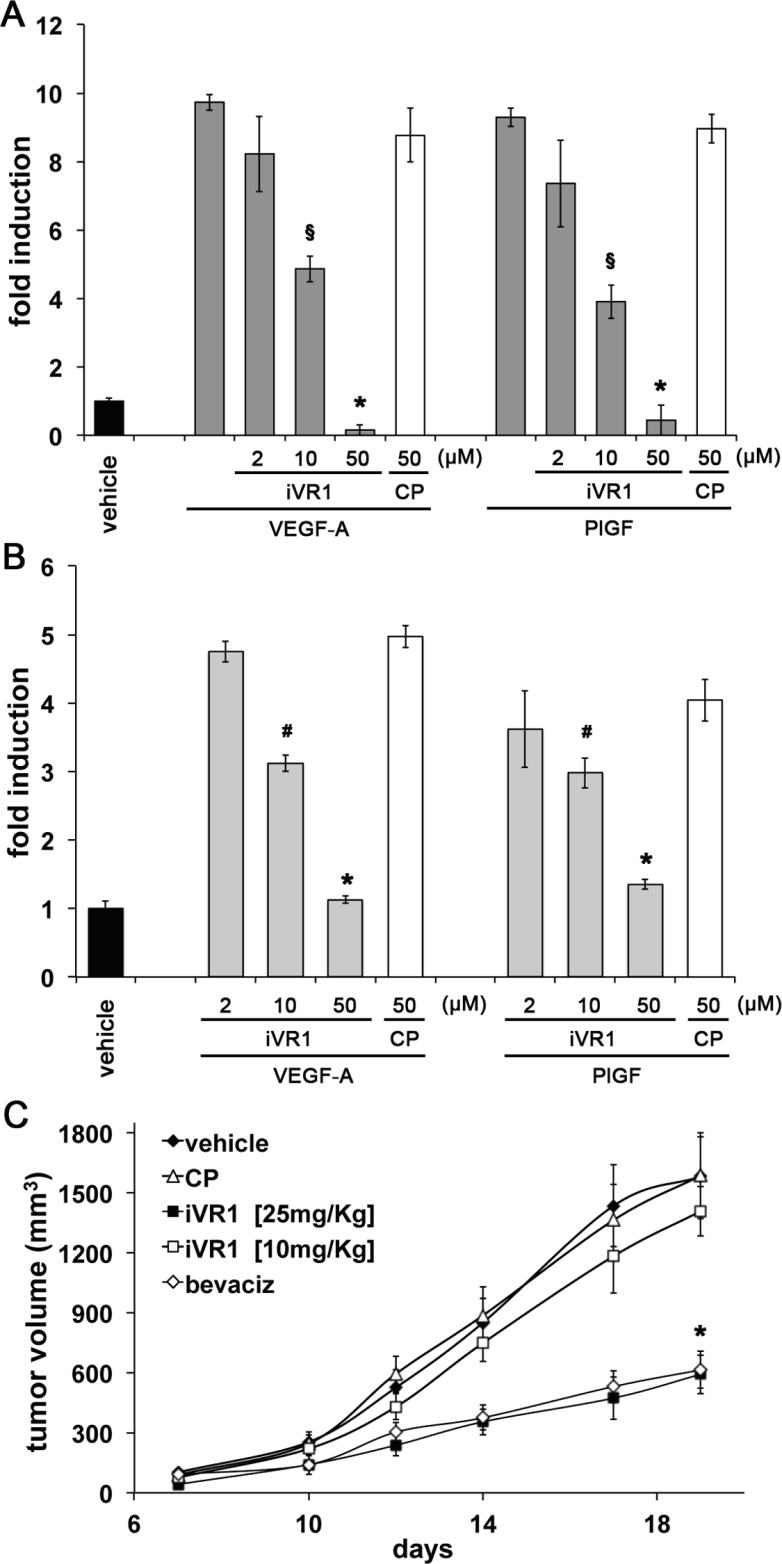
Dose-dependent inhibition of macrophage migration and xenograft tumor growth exerted by iVR1 iVR1, but not CP, was able to inhibit migration of mouse RAW264.7 macrophages **(A)** and ex vivo peritoneal macrophages **(B)** stimulated by VEGF-A or PlGF, in a dose-dependent manner. Data are expressed as fold induction with respect to vehicle (DMSO). A, *p < 0.0005 and ^§^*p* < 0.001 versus CP and VEGF-A or PlGF. B, *p < 0.0005 and ^§^*p* < 0.05 versus CP. **(C)** Dose-dependent inhibition of HCT-116 tumor growth exerted by iVR1 delivered at 10 or 25 mg/kg each other day. Bevacizumab (bevaciz) was delivered at 5 mg/kg two times at week. *N* = 7, *p < 0.001 versus vehicle and CP.

The *in vivo* effects of iVR1 on cell recruitment were confirmed *in vitro* by inhibition of VEGF-A and PlGF-induced migration of murine RAW 247.6 macrophages and *ex vivo* isolated peritoneal macrophages. Growth factors stimulated to similar extents cell migration, which was inhibited by iVR1 in a dose-dependent manner already at 10 μg/ml (Figure 5A, 5B).

In the attempt to optimize the dosage of iVR1, we treated mice bearing xenograft tumors with decreasing doses of compound (25 mg/kg and 10 mg/kg), while control mice were treated with vehicle and CP or bevacizumab at the dosages previously used (Figure 5C). iVR1 at 25 mg/kg still inhibited tumor growth as much as bevacizumab, while at 10 mg/kg no significant inhibition was observed. In subsequent experiments iVR1 thereby was delivered at 25 mg/kg.

### iVR1 and irinotecan synergistically inhibited CRC *in vivo* growth

**Figure 6:**
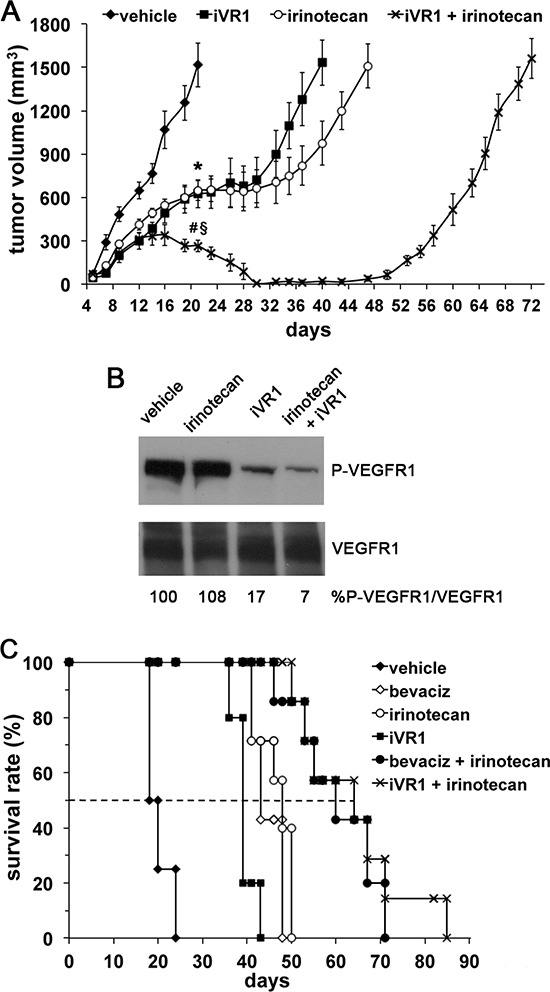
iVR1 showed a synergic effect in combination with irinotecan **(A)**, iVR1 (25 mg/kg, each other day) and irinotecan (50 mg/kg once at week) were delivered alone or in combination starting at day 5 from cell inoculation and for 24 days. TV was measured three times a week and data are represented as the mean ± SEM (*N* = 7). At day 21: *p < 0.005 for iVR1 and irinotecan compared to vehicle, ^§^*p* < 0.0001 versus vehicle, ^#^*p* < 0.005 vs iVR1 and irinotecan. **(B)**, top, western blot analysis of VEGFR1 phosphorylation performed on mixed matching amounts of protein extracts belonging to the same experimental tumor group. Low, normalization with anti-VEGFR1 antibody performed on the same filter. The values of densitometry analyses are shown. Values (in percentages) were calculated as the ratio of degree of receptor phosphorylation with respect to the total receptor amounts. The value of 100 has been arbitrarily assigned to vehicle. **(C)**, Kaplan-Meier survival curves of iVR1, irinorecan and bevacizumab (bevaciz, 5 mg/Kg two times a week) treatments, alone or in combination. Drugs were delivered as in A. Dashed line indicates 50% of survival. Bevaciz, irinotecan or iVR1 treatments were significantly lower compared to vehicle (*p* < 0.01) as well as the two combination treatments, iVR1 plus irinotecan and bevaciz plus irinotecan (*p* < 0.001).

Among systemic treatments clinically approved for mCRC patients, irinotecan and bevacizumab containing regimens are largely used for their efficacy. Therefore, we evaluated if iVR1 activity might synergize with irinotecan, a camptotecin-based inhibitor of topoisomerase I [32]. Drugs were delivered starting at day 5 from cells inoculation and the combination treatment was compared to single treatments or vehicle (Figure 6A). At day 21 from cell inoculation, tumor growth inhibition induced by iVR1 was similar to that induced by irinotecan and both were significantly greater compared to vehicle (−58.3%, *p* < 0.005). Strikingly, the iVR1-irinotecan combination induced a very powerful inhibition of tumor growth already at day 21 (−82.1%, *p* < 0.0001 versus vehicle and −58.2%, *p* < 0.005, versus iVR1 and irinotecan alone), causing a progressive reduction of the tumors volume starting at day 16. Since we observed an almost total regression of tumors in the combination group, treatments were stopped seven days later. Indeed, at day 28, only two out of seven tumors were still detectable and for the next 20 days tumors were not measurable, with a delay in tumor relapse of about 50 days compared to vehicle-treated tumors. iVR1-treated tumors relapsed more rapidly than the irinotecan-treated ones, both reaching 1500 – 1600 mm^3^ average volume 20 and 30 days later, respectively, than vehicle-treated tumors (Figure 6A).

Therefore, we evaluated the inhibition of VEGFR1 phosphorylation after delivery of iVR1 at 25 mg/kg. While irinotecan, as expected, did not affect VEGFR1 phosphorylation, in iVR1 and iVR1 plus irinotecan-treated tumors, a potent inhibition of VEGFR1 was observed. Inhibition was equivalent to that achieved delivering iVR1 at 50 mg/kg, thus indicating that even at 25 mg/kg iVR1 affords a powerful anti-VEGFR1 activity *in vivo* (Figure 6B).

In order to evaluate the impact of treatments on the survival rate of tumor-bearing mice, and to compare the effect of iVR1-irinotecan combination with that of bevacizumab-irinotecan, we performed a further experiment in which animals were treated with vehicle, irinotecan, iVR1, bevacizumab, iVR1-irinotecan and bevacizumab-irinotecan. Drugs were delivered, as for previous experiment, for 24 days starting from day 5 from cell inoculation and animals were sacrificed when tumors reached a volume close to 2000 mm^3^. Kaplan-Meier distribution is reported in Figure 6C. Treatments with bevacizumab, irinotecan or iVR1 likewise determined a median increase of survival comprised between 20 and 28 days, compared to vehicle-treated mice. Again, the combination iVR1-irinotecan confirmed its synergic effect showing an activity fully comparable to that of the bevacizumab-irinotecan combination with a survival median increase of more than 40 days, compared to vehicle-treated mice.

Remarkably, no apparent signs of toxicity were observable after delivering iVR1 at 50 mg/kg for 14 days and at 25 mg/kg for 80 days, administered each other day. Also, body weight was recorded and no differences were observable in iVR1 treated mice as compared to vehicle or other controls (data not shown).

Collectively, these data confirmed the powerful therapeutic potential of iVR1 alone and in combination with irinotecan, which resulted comparable to that of bevacizumab alone or in combination regimen.

### iVR1 prevented the development of CRC lung metastases in mice

VEGFR1 positive hematopoietic progenitors have been involved in metastatization process [22]. We thus investigated the effect of iVR1 in an artificial model of metastatization. HCT-116 cells were injected in the tail vein of nude mice and treatments with bevacizumab, iVR1, CP or vehicle (*N* = 5 per group) started immediately after cell injection, following the same schedule adopted for the combination and survival experiments. DNA was extracted from mice lungs to quantify human *Alu* sequences by qRT-PCR (Table 1 and Supplementary Figure 1). *Alu* sequences were well detectable at similar levels in the lungs of vehicle and CP treated mice. Bevacizumab induced a strong reduction compared to vehicle and CP whereas in iVR1-treated mice the quantity of *Alu* sequences was barely detectable, suggesting that iVR1 was also active in preventing the transmigration of colorectal cancer cells from blood circulation to lung.

**Table 1:** Quantification of human *Alu* sequences in DNA extracted from mice lungs

Standard curve		Sample analysis		
Human genomic DNA [ng]	CTm ± SD	Sample	CTm ± SD	ng
200	19.02 ± 0.45	Vehicle	19.99 ± 0.20	121.87 ± 1.22
20	22.67 ± 0.83	CP	19.70 ± 0.36	145.91 ± 2.67
2	26.37 ± 0.52	bevaciz	22.68 ± 0.04	22.93* ± 0.04
0.2	29.75 ± 085	iVR1	30.05 ± 0.87	0.24* ± 0.01
0.02	33.99 ± 1.03			

Standard concentrations of human genomic DNA were used as template for qRT-PCR to amplify *Alu* sequences. For each concentration analyzed, the average values of cycle threshold (CTm) ± SD are reported (each point in triplicate) and a standard curve was constructed. Genomic DNA extracted from lungs of mice treated with vehicle, iVR1, CP and bevacizumab (bevaciz) (*N* = 5 per group) were amplified (see Supplementary Figure 1). Based on the average value of cycle threshold (CTm) obtained, the quantity of human *Alu* sequences were extrapolated by comparison with the standard curve, using exponential regression.

* *p* < 0.0001 versus vehicle and CP.

## DISCUSSION

The role of VEGFR1 in pathological angiogenesis and its participation to the angiogenic switch and metastatic process are still under debate [3, 21, 22, 33]. We report a potent anti-tumor, anti-angiogenic and anti-metastatic activity of the antagonist iVR1, which supports the important role played by VEGFR1 in tumor and, more in general, in pathological angiogenesis.

Among the several non-protein VEGFR1 antagonists so far described [23, 25–27, 34, 35] those with proven *in vivo* tumor inhibition activity are a ribozyme, the DNAzyme DT-18, the hexapeptide GNQWFI and the 20-aminoacid peptide named BP-1 [23, 25–27]. None of these molecules have been so far tested in combination with chemotherapeutics nor assessment of survival rate has been reported. Therefore, iVR1 is the first VEGFR1 non-protein antagonist for which extensive characterization as anti-tumor agent is reported and is also the first showing potent inhibition of VEGFR1 phosphorylation *in vivo*. This property, together with the effects observed on tumor vascularization, on inflammatory cells infiltration and on vessel stabilization, suggests the following considerations. The partial inhibition of VEGFR1 activation observed with mAbs anti-PlGF had the lowest effect on tumor growth and neoangiogenesis inhibition, as expected by the selective blockade of the PlGF/VEGFR1 axis. A similar reduction of VEGFR1 phosphorylation induced by bevacizumab, but associated with the inhibition of VEGFR2 activation through the blockade of VEGF-A, determined a much stronger decrease of tumor growth, neovascularization, and monocyte macrophage recruitment. Interestingly, the nearly fully block of VEGFR1 activation promoted by iVR1 translated into effects similar to those produced by bevacizumab, at least in terms of tumor growth and mural cells recruitment. Of note, while bevacizumab caused the highest reduction of neovessel formation, iVR1 determined the highest reduction of monocyte-macrophages infiltration. We hypothesize that the strong inhibition of monocyte-macrophage recruitment at neo-angiogenic site, where they sustain and fuel pathological angiogenesis [30, 31], together with the potential inhibiting effects on the recruitment of others VEGFR1-positive bone marrow derived precursors, seemingly compensate for the lower effect on neovessel formation, as compared to bevacizumab activity.

Another potent VEGFR1 inhibitor is the mAb IMC-18F1 (icrucumab, Eli Lilly and Company), which in preclinical models of breast cancer inhibits tumor xenograft growth and synergizes with different chemotherapeutics, including cyclophosphamide or doxorubicin [16, 24]. IMC-18F1 is currently in phase 2 of clinical trials, for unresectable locally advanced or metastatic breast cancer in combination with capecitabine (http://clinicaltrials.gov identifier NCT01234402), and as second-line therapy in combination with docetaxel, for locally advanced or metastatic transitional cell carcinoma of the bladder, urethra, ureter, or renal pelvis (http://clinicaltrials.gov identifier NCT01282463). Interestingly, preclinical studies showed that when used in combination with the mAb IMC-MF1, specific for mouse VEGFR1, IMC-18F1 enhanced the anti-tumor efficacy [24]. These findings clearly indicated the relevance of blocking either human VEGFR1, expressed by tumor cells, and mouse VEGFR1, expressed on endogenous cells, which both contribute to tumor formation. In this regard, a major feature of iVR1 accounting for the potent activity showed in xenograft models is the ability to inhibit both human and mouse VEGFR1.

Differently from the current anti-angiogenic drugs (bevacizumab, aflibercept and TK inhibitors) used to treat cancer patients, iVR1 does not interfere with the VEGFA/VEGFR2 pathway. Although it is widely accepted that the blockade of VEGFA/VEGFR2 is crucial for inhibiting neovessel formation, mainly for the direct effects on endothelial cells, the impressive anti-tumor activity achieved with the irinotecan-iVR1 combination in CRC models, and the resulting outstanding survival rate comparable to that of irinotecan-bevacizumab combination, strengthen the concept that a potent and selective VEGFR1 inhibition produces therapeutic effects comparable to those caused by the blockade of VEGF-A. This is probably mainly due to the potent inhibition of the recruitment of non-endothelial VEGFR1 positive cells involved in neo-vessels formation. Moreover, VEGFR1 blockade may be advantageous in terms of toxicity. As indeed previously reported, blocking VEGFR1 or its specific ligand PlGF is less detrimental compared to VEGF-A blockade [36, 37], which, given the vital role of VEGF-A/VEGFR2 pathway in the physiological homeostasis of vessels [38], is responsible of the several side effects observed not only in cancer, but also in ocular neovascular diseases [6, 7].

Concerning the anti-metastatic activity of iVR1, this is in line with that exerted by the other VEGFR1 inhibitors previously mentioned, the ribozyme, the hexapeptide GNQWFI [23, 25], and the peptide BP-1 [26]. Based on our data, we hypothesize that the ability to prevent the activation and migration of VEGFR1 positive bone marrow precursors necessary for pre-metastatic niche formation [22], might represent one of the mechanisms by which iVR1 is able to fully prevent the transmigration of cancer cell from blood circulation to the lungs.

iVR1 is also able to potently reduce laser-induced CNV, the pre-clinical model for the age-related macular degeneration (AMD) condition. AMD is a VEGF-A-driven disease and currently patients are treated with ranibizumab or aflibercept, approved by FDA, or with bevacizumab off label [39]. Therefore, the anti-angiogenic activity of iVR1 may be also explored in different pathological contexts where angiogenesis is involved.

Altogether, our results strongly support the concept that targeting VEGFR1 with selective inhibitors has a huge therapeutic potential in cancer, and more in general, in angiogenesis-driven diseases. Despite the micromolar affinity for VEGFR1, the outstanding anti-angiogenic properties, the anti-tumor features similar to those of bevacizumab, especially when it is used in combination with chemotherapeutics, and the preliminary observations supportive of a seemingly very low toxicity, makes iVR1 a very good candidate for further development. Further assessment of toxicity, drug metabolism and pharmacokinetics will be undertaken together with chemical modifications to improve potency, solubility and eventually absorption and distribution, to further improve the therapeutic index of this very promising compound.

## MATERIALS AND METHODS

### iVR1 peptide preparation

iVR1 and control peptides were synthesized as previously described [28]. For the *in vivo* delivery, we used as vehicle 50% polyethylene glycol (PEG) 400, 50% sterile water. Two hours before injection, peptides were suspended in PEG400/water by magnetic stirring at 50°C in sterilized glass vials. We obtained a stable and high homogeneous suspension containing up to 20 mg/ml of peptides.

### Cell culture

CT26 (murine colon carcinoma), RAW 246.7 (murine macrophage) and HCT-116 (human colorectal carcinoma) cell lines, all from American Type Culture Collection, were grown in RPMI 1640, DMEM and McCoy's medium (Euroclone) respectively, supplemented with 10% inactivated Fetal Bovine Serum, 2 mM glutamine and standard concentration of antibiotics. Mouse macrophages were isolated from peritoneum of C57BL/6J mice by intraperitoneal injection of 1 mL of 3% Brewer thioglycollate Medium (Sigma). After 4 days mice were sacrificed and peritoneal lavage with cold-ice D-PBS 20% FBS were performed to recover macrophages, which were stained with biotinylated rat anti mouse F4/80 (1:50; Serotec) and isolated using anti-biotin MicroBeads and MS Separation columns (Miltenyi Biotec).

### Cell migration

Ex vivo isolated macrophages or starved RAW246.7 cells (40000 cells/well) were placed into the upper chamber of a 24-multiwell insert system with 5-μm pore size polycarbonate filter (Corning). Cell migration was stimulated with VEGF-A or PLGF-1 (100 ng/mL) added to the starvation medium into the lower chamber, in presence of iVR1 (2 μM, 10 μM or 50 μM) or CP (50 μM). After 24 h for RAW246.7, or 6 h for peritoneal macrophage, cells on the top of the filter were removed and those on the bottom side were stained with DAPI. Images were recorded on Leica DMI 6000 microscope equipped with Hamamatsu Orca R2 camera. Single cells were counted using Tile Scan Macro of LAS AF Software (Leica).

### Animals

Balb/c mice or CD1 nude athymic mice, were purchased from Charles River. C57Bl/6J mice were purchased from The Jackson Laboratory. Animal experiments were in accordance with European directives no. 2010/63/UE and with Italian directives D.L. 26/2014, and with the guidelines of the University of Kentucky Institutional Animal Care and Use Committee. For all procedures, anesthesia was performed by intraperitoneal injection of 100 mg/kg ketamine hydrochloride and 10 mg/kg xylazine.

### Syngeneic and xenograft tumor models

7- to 8-week-old male Balb/c mice for syngeneic tumors model, or CD1 nude athymic mice for xenografts tumor model, were injected subcutaneously with 1 × 10^6^ CT26 or 4 × 10^6^ HCT-116 cells into the right flank, respectively. Tumor volume (mm^3^) was quantified three times a week by measuring tumor shortest (d) and longest (D) diameters with an electronic caliper, using the formula D x d^2^/2. For all experiments, when tumors became measurable (by day 6 or 7 from cells injection, 50–100 mm^3^) mice were randomly divided (*n* = 7). Treatments were performed by intraperitoneal injections (max volume 100 μl) with the following schedules: vehicle PEG400/H_2_O 1:1, iVR1, 10 to 50 mg/kg and control peptide (CP), 50 mg/kg, each other day; monoclonal antibodies anti-PlGF 5D11D4 and 16D3 (Thrombogenics), 25 mg/kg, or bevacizumab (Genentech), 5 mg/kg, twice a week; irinotecan 50 mg/kg once a week. Mice weight was recorded at each tumor measurement. For ethical reasons, mice were sacrificed when tumor reached a volume between 1500 and 2000 mm^3^. The highest dose of iVR1 at 50 mg/kg was chosen on the basis of the estimated IC_50_ of 6–10 μM [28].

### Artificial metastasis assay and quantification of human *Alu* sequences

7- to 8-week-old male CD1 nude athymic mice were anesthetized and injected with 3 × 10^5^ HCT-116 cells via tail vein. Treatments by day zero and for 24 days, were performed with vehicle, CP and iVR1 (50 mg/kg), or bevacizumab, following the schedule reported before. Mice were sacrificed on day 25 and genomic DNA was extracted from all the lobes of mice lungs using QIAamp DNA Mini Kit (Qiagen). The concentration of double-stranded DNA was determined using BIORAD Experion and 1K Experion DNA Kit (Biorad). To quantify human *Alu* sequences in DNA extracted from mice lungs, specific primers reported by Schneider et al. [40] were used to perform qRT-PCR as previously described [41].

### Tumor protein extracts and western blot analysis

Frozen tumor samples were disrupted with a Tissue-Lyser (Qiagen), 100 mg in 300 μL of lysis buffer (10 mM Tris-HCl pH 8, 150 mM NaCl, 1% Triton-X 100, 0.1% SDS, 0.5% Na-Deossycolate, 0.2% NaN3) supplemented with protease inhibitors (Roche), for 5 minutes at 3000 rpm. The supernatants were recovered and stored at −80°C. Western blot analyses on mixed matching amounts of extracts belonging to the same experimental tumor group were performed as previously described [42].

### Immunohistochemical analyses

10 μm-thick cryiopreserved tumors sections were fixed with PFA 4% and incubated overnight at 4°C with the following primary antibodies: rat anti-mouse PECAM-1 (anti-CD31; 1:1000; BD Pharmingen), rat anti-mouse F4/80 (1:50; Serotec) and anti-mouse smooth muscle α-actin (1:1000; DAKO). The staining procedure was continued using specific secondary biotinylated antibody (all from DAKO). Slides were counterstained with hematoxylin. Images were recorded with a digital camera Leica. Densitometric analyses were performed with QwinPro software (Leica). Quantifications were performed on five optical fields for each tumor.

### Choroid neo-vascularization

Laser photocoagulation procedure and CNV volumes determination were performed as previously described [43]. Immediately after laser application, 10 μg (4.2 nmol) and 50 μg (21 nmol) of iVR1 or 50μg of CP, in 1 μl of vehicle (DMSO), were injected intravitreally with a 33-gauge Exmire microsyringe (Ito Corporation) (*N* = 4 per group). As control, 2 ng of polyclonal anti-mouse VEGF-A antibody (R&D Systems) in 1 μl PBS were injected.

### Statistical analysis

Results are expressed as mean ± standard error of the mean (SEM), with *P* values < 0.05 considered statistically significant. Differences among groups were compared by the Student's *t* test (two-tailed) or 1-way ANOVA. Tukey HD test was used as a post hoc test to identify which group differences account for the significant overall ANOVA. Log-rank test was performed for Kaplan-Meier survival curves statistical analysis. All calculations were carried out using SPSS statistical package (vers14.1; SPSS, Inc., Chicago, IL).

## SUPPLEMENTAL FIGURES


