# Supplementary material for: Powerful anti-tumor and anti-angiogenic activity of a new anti-vascular endothelial growth factor receptor 1 peptide in colorectal cancer models
Source: Oncotarget. 2015 Mar 25;6(12):10563–76. doi: 10.18632/oncotarget.3384 (PMC4496375; doi:10.18632/oncotarget.3384)
Supplement: Supplementary file 1 [file oncotarget-06-10563-s001.pdf]

SUPPLEMENTARY FIGURE

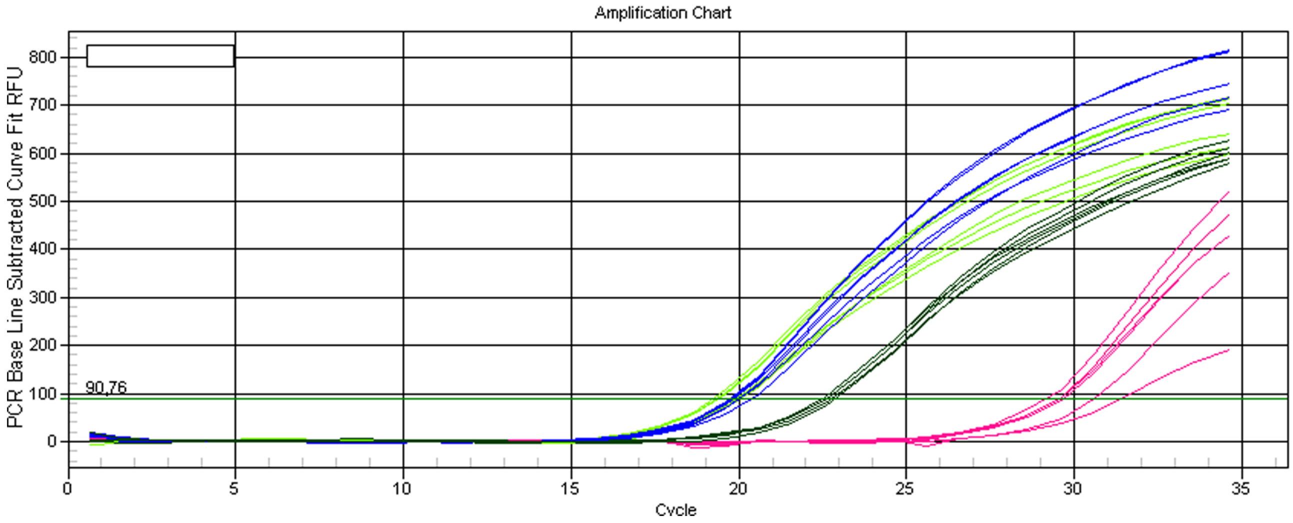

Supplementary Figure 1: Amplification plot of Alu sequences using as template genomic DNA extracted fom lungs of treated mice (*N* = 5 per group). Vehicle, blue curves (mean CT 19,99); CP, green curves (mean CT 19,70); Bevacizumab, dark green curves (mean CT 22,68) and iVR1, pink curves (mean CT 30,05). Each amplification was carried out in duplicate.
